# Synteny analysis in Rosids with a walnut physical map reveals slow genome evolution in long-lived woody perennials

**DOI:** 10.1186/s12864-015-1906-5

**Published:** 2015-09-17

**Authors:** Ming-Cheng Luo, Frank M. You, Pingchuan Li, Ji-Rui Wang, Tingting Zhu, Abhaya M. Dandekar, Charles A. Leslie, Mallikarjuna Aradhya, Patrick E. McGuire, Jan Dvorak

**Affiliations:** Department of Plant Sciences, University of California, Davis, CA USA; Cereal Research Centre, Agriculture and Agri-Food Canada, Morden, Canada; United States Department of Agriculture-Agricultural Research Service Clonal Repository, Davis, CA USA; Triticeae Research Institute, Sichuan Agricultural University, Wenjiang, China

**Keywords:** Fagales, Angiosperm, Dysploidy, Polyploidy, Juglandaceae, Recombination rate, Single nucleotide polymorphism, Molecular clock

## Abstract

**Background:**

Mutations often accompany DNA replication. Since there may be fewer cell cycles per year in the germlines of long-lived than short-lived angiosperms, the genomes of long-lived angiosperms may be diverging more slowly than those of short-lived angiosperms. Here we test this hypothesis.

**Results:**

We first constructed a genetic map for walnut, a woody perennial. All linkage groups were short, and recombination rates were greatly reduced in the centromeric regions. We then used the genetic map to construct a walnut bacterial artificial chromosome (BAC) clone-based physical map, which contained 15,203 exonic BAC-end sequences, and quantified with it synteny between the walnut genome and genomes of three long-lived woody perennials, *Vitis vinifera*, *Populus trichocarpa*, and *Malus domestica*, and three short-lived herbs, *Cucumis sativus*, *Medicago truncatula,* and *Fragaria vesca*. Each measure of synteny we used showed that the genomes of woody perennials were less diverged from the walnut genome than those of herbs. We also estimated the nucleotide substitution rate at silent codon positions in the walnut lineage. It was one-fifth and one-sixth of published nucleotide substitution rates in the *Medicago* and *Arabidopsis* lineages, respectively. We uncovered a whole-genome duplication in the walnut lineage, dated it to the neighborhood of the Cretaceous-Tertiary boundary, and allocated the 16 walnut chromosomes into eight homoeologous pairs. We pointed out that during polyploidy-dysploidy cycles, the dominant tendency is to reduce the chromosome number.

**Conclusion:**

Slow rates of nucleotide substitution are accompanied by slow rates of synteny erosion during genome divergence in woody perennials.

**Electronic supplementary material:**

The online version of this article (doi:10.1186/s12864-015-1906-5) contains supplementary material, which is available to authorized users.

## Background

Most mutations originate during DNA replication. The divergence of nucleotide sequences should therefore be related to the number of germline cell divisions per unit of time rather than to time alone [1]. Because the nucleotide substitution rate (molecular clock) is expressed per year, molecular clock should tick slower in taxonomic groups with long life-cycle length, although other factors may modify a clock’s rate [2–4].

In angiosperms, the number of cell divisions in the germline may differ among different species even if their life-cycle lengths were similar due to differences in plant development and reproduction. It might therefore be difficult to detect the relationship between life-cycle length and the rate of molecular clock in angiosperms. Contradictory evidence was initially reported [5–10] but an extensive study employing related taxa differing in life-cycle lengths provided strong evidence supporting this relationship [11].

Another facet of genomic change is the erosion of synteny between the genomes of related species. Synteny erosion is caused by the accumulation of duplications, deletions, inversions, translocations, and transpositions of chromosomal segments of various lengths or individual genes and their fragments. These structural changes perturb the sequence of genes along chromosomes. Gene duplications and deletions, sometimes referred to as gene copy number variation, may lead to the evolution of new genes [12]. Gene duplications and deletions and the evolution of new genes is an important evolutionary strategy in angiosperms [13].

The rates of gene duplication and deletion were shown to vary extensively among lineages in the grass family and lead to variation in the number of genes in grass genomes [14]. The causes of this variation are unclear. Genome size and the activity of transposable elements (TEs) were speculated to play roles [14–17]. TEs cause DNA rearrangements [18] and gene and gene fragment duplications [19–24]. TE transposition is often mobilized by DNA replication [25]. We therefore hypothesize that the rates of synteny erosion, like those of the molecular clock, may depend on the life-cycle length. A report [26] that synteny of the grape genome with that of the poplar, a long-lived woody perennial, is more conserved than with that of *Arabidopsis thaliana,* a short-lived ephemeral herb, is consistent with this hypothesis.

To study this relationship, we quantify here synteny in the “nitrogen-fixing” clade and two related clades of angiosperms. The nitrogen-fixing clade includes orders Fabales, Rosales, Cucurbitales, and Fagales [27]. The clade contains both woody perennials and herbs with reference-quality genome sequences, facilitating the study of synteny. We selected among them the genome sequences of *Medicago truncatula* (Fabales) [28], apple (*Malus domestica*) (Rosales) [29], strawberry (*Fragaria vesca*) (Rosales) [30], and cucumber (*Cucumis sativus*) (Cucurbitales) [31]. No species with a reference-quality genome sequence exists in Fagales. Previous studies demonstrated that in the absence of a reference quality genome sequence synteny can be effectively assessed using a dense comparative genetic map [15] or comparative physical map [17, 32, 33]. We therefore develop here a comparative physical map for Persian (English) walnut (*Juglans regia*), a woody perennial economically important for nuts and timber. Walnut belongs to the family Juglandaceae. Juglandaceae and seven other families make up the order Fagales [34].

Fagales with the remaining orders of the nitrogen-fixing clade are members of the clade Fabidae (syn. Eurosids I). Fabidae and the sister clade Malvidae (syn. Eurosids II) form the Rosid clade, which contains about a quarter of all angiosperms [27].

In addition to species of the nitrogen-fixing clade we include in our study the genomes of two more long-lived woody perennials, the grape (*Vitis vinifera*) and poplar (*Populus trichocarpa*). A reference-quality genome sequence exists for both species [8, 26]. Poplar is a member of Malpighiales, which is a sister clade of the nitrogen-fixing clade within Fabidae [35] or a clade of Malvidae [30]. Grape is a member of Vitales, which is either a sister clade to Fabidae and Malvidae within Rosids [27] or is basal to the Rosid clade [36–39].

The analysis of the *V. vinifera* genome sequence revealed a whole genome triplication (γ triplication) [26], which was estimated to have taken place about 117 million years (MY) ago and was proposed to predate the radiation of the Rosid clade [40]. Because the grape genome does not reveal any other whole genome duplication (WGD) we use the grape genome as a baseline reference in the search for WGDs. The γ triplication was reported to be detectable in the apple genome [29] but no conclusive evidence for it was found in the strawberry genome [30], even though apple and strawberry belong to the same family. Two WGDs were detected in the poplar genome [8], one recent, named “salicoid”, and one more ancient. It is not entirely clear whether the older WGD corresponds to the γ triplication detected in the grape genome.

To initiate the construction of the walnut comparative physical map, we previously developed two libraries of bacterial artificial chromosome (BAC) clones for cv ‘Chandler’, a clonally propagated heterozygous walnut cultivar, and sequenced one end of 48,218 BAC clones [41]. We filtered the BAC-end sequences (BES) for coding sequences (henceforth cdBES), aligned cdBES with multiple walnut genome equivalents of Chandler next generation sequence (NGS) reads, identified single nucleotide polymorphisms (SNPs) [42], and used them to design a 6 K Infinium SNP assay for walnut [42].

Here we deploy these resources in the construction of a walnut comparative physical map. Because the walnut physical map contains only a subset of the total number of genes in the walnut genome, we limit the synteny comparisons to that subset and employ the walnut physical map in all genome comparisons. Using this strategy, all synteny quantifications we make are comparable because they all use the same set of coding sequences.

## Results

### Genetic map

The standard approach to construct a BAC-based physical map is to construct a dense genetic map, and used it as a backbone for anchoring and ordering on it BAC contigs. We genotyped a walnut mapping population of 425 F_1_ trees from a cross of Chandler with ‘Idaho’, two heterozygous clonally propagated walnut scion cultivars, with the walnut 6 K Infinium SNP iSelect assay [42]. Only markers that segregated in Chandler and were homozygous in Idaho were used in the construction of the genetic map. The map was therefore a female-backcross map. We mapped 1,525 SNP markers (Additional file 1) into 16 linkage groups (LG) corresponding to the 16 walnut chromosomes (Figure S1 in Additional file 2). The lengths of the LGs ranged from 37.7 cM for LG15 to 97.3 cM for LG7 (Table 1). The average length of a LG was 65.4 cM and the total length of the genetic map was 1,049.5 cM (Table 1).

**Table 1:** Characteristics of the walnut genetic and physical maps

Chrom.	Mapped SNP markers (no.)	Genetic map length (cM)	Anchored BAC contigs (no.)	cdBES (no.)	MTP length (Mb)	Location of the centromeric interval on the physical map (Mb)
Jr1	122	79.9	35	1441	63.398	38.4-48.4
Jr2	92	70.5	26	968	48.622	13.2-19.6
Jr3	139	64.3	40	1134	56.126	44.3-46.3
Jr4	112	53.7	35	1000	52.028	43.8-44.8
Jr5	48	45.7	20	478	26.040	12.0-16.9
Jr6	116	60.2	37	1020	56.732	24.4-31.6
Jr7	130	97.3	39	1355	61.010	32.3-34.1
Jr8	78	93.5	18	736	36.629	25.8-31.3
Jr9	56	69.3	19	615	27.927	7.5-11.3
Jr10	135	63.5	55	1253	71.659	34.2-39.5
Jr11	125	66.5	32	1167	54.433	24.0.-30.0
Jr12	92	60.5	24	880	42.027	11.0-13.6
Jr13	94	70.0	29	1000	46.491	38.5-40.8
Jr14	85	57.6	18	1033	44.439	27.6-34.6
Jr15	13	37.7	6	203	9.573	?
Jr16	88	59.3	21	920	38.935	14.7-20.2
Total	1525	1049.5	454	15203	736.070	

### Physical map

We fingerprinted 124,890 Chandler BAC clones with the SNaPshot high-information-content fingerprint (HICF) technology [43] modified as described by Gu et al. [44]. A total of 122,274 clones (97.91 %) contained usable fingerprints. After removing contaminated clones, clones with substandard fingerprints, and clones possessing small inserts, we used 113,074 clone fingerprints (92.5 %) for contig assembly. The initial assembly resulted in 916 contigs containing 108,233 BAC clones (Table 2). The remaining 4,841 clones were singletons. The average contig length was 1.128 Mb, N50 = 2.083 Mb, and L50 = 154. From the available 48,218 Chandler BES [41] [GenBank accession numbers from HR182515 to HR231850] we selected cdBES [42]. We used the cdBES containing SNP marker sequences for anchoring BAC contigs onto the genetic map. We manually examined the consistency of the order of the markers along each BAC contig and along the genetic map and edited problematic contigs. That usually meant manual disjoining of a problematic contig into two or more contigs. Contig editing consequently increased the total number of BAC contigs from 916 to 1,031 (Table 2 and Additional file 3). The average contig length and N50 length slightly decreased while L50 increased (Table 2). Of the 1,031 edited contigs, 562 were anchored on the genetic map. We generated a contiguous sequence of consensus bands (CB) across each BAC contig with FPC [45] and converted physical maps in terms of CB units into physical maps in terms of bp using the relationship 1 CB unit = 1.258 Kb, which we estimated with FPC. We estimated the total length of the physical map across the 562 anchored contigs to be 859.6 Mb, accounting for 77 % of the total contig length. The unanchored 23 % of the genome were short BAC contigs (Additional file 3).

**Table 2:** BAC contig statistics before and after manual editing

	No. contigs	Average no. clones per contig	Average contig length (Kb)	N50 length (Kb)	L50
Before editing	916	118.16	1128.15	2083	154
After editing	1031	105.18	1018.07	1855	181

Chandler is a heterozygous clone, and in some contigs FPC assembled the two Chandler haplotypes separately, nesting one contig into another. The merodiploidy of the physical map would double the length of the map in those regions and would create artifacts in map applications. We therefore removed the shorter of the nested contigs from the physical map (Additional file 3). A total of 113 nested contigs were removed, which reduced the number of anchored contigs from 562 to 454 and the length of the physical map from 859.6 Mb to 736.1 Mb (Table 1), shortening it by 14.4 %. Although the physical maps contained 438 gaps between contigs, the map contains a large portion of the genome as suggested by the fact that the estimated total length of the physical map anchored on the genetic map, about 736.1 Mb, was close to the estimated size of the walnut genome, 606 Mb (Horjales et al. 2003 in http://data.kew.org/cvalues/).

A total of 15,203 BAC clones in the 454 contigs contained cdBES, which we annotated (Excel Table S3 in Additional file 4). The cdBES are listed in column E (heading “BAC”) of the Excel Table S3 in Additional file 4. We determined the beginning of each BAC clone containing a cdBES on the CB map of each contig. The sequence (measured in Kb) of these cdBES along the BAC contigs and along each of the 16 walnut chromosomes, disregarding the gaps between contigs, generated a comparative physical map of each walnut chromosome.

Since each physical map was anchored on a linkage map, the physical maps Jr1 to Jr16 corresponded to LG1 to LG16. Because only a single end of a BAC clone was sequenced [41], only a single cdBES could be present in a BAC clone. Since we did not know at which end of a BAC clone the cdBES was located, ordering of cdBES along the physical map had an error of the length of one BAC clone, 100 to 200 kb. For this reason, and the inadvertent anchoring of BAC clones on the genetic map with paralogous genes, the order of neighboring markers on the genetic map (Additional file 1) was occasionally inverted on the physical map (Additional file 4). The numbers of cdBES per physical map ranged from 203 along physical map Jr15 to 1,441 along physical map Jr1 (Table 1 and Additional file 4).

### Recombination rates along walnut chromosomes

**Fig. 1:**
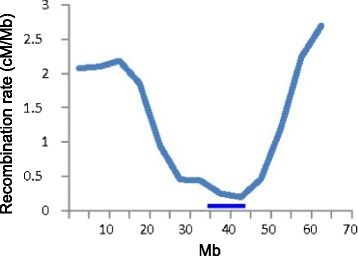
Recombination rates along chromosome Jr1. The horizontal axis is the physical map in Mb of Jr1 and the vertical axis is recombination rate in cM/Mb. The horizontal bar marks the location of a gap in synteny between the Jr1 physical map and the grape, poplar, apple, cucumber, *Medicago truncatula*, and strawberry pseudomolecules. We suggest that the collocation of the recombination rate global minimum and the synteny gap marks the Jr1 centromeric region

Using a sliding window of 5 Mb, we computed recombination rates, expressed as cM/Mb, for 15 of the 16 walnut physical maps; that of Jr15 was too short (9.57 Mb) for a meaningful assessment of recombination rates. Recombination rates were highest in the subterminal regions. The average of these local maxima was 2.5 ± 0.74 cM/Mb (mean and standard deviation, respectively). A graph illustrating recombination rates along physical map Jr1 is shown in Fig. 1; graphs for all physical maps except for that of Jr15 are in Additional file 5. All 15 investigated physical maps had a single global minimum with an average recombination rate of 0.63 ± 0.33 cM/Mb.

### Synteny and its quantification

We searched for homology between the 15,203 cdBES ordered along the 16 walnut (Jr) physical maps and genes on the grape (Vv), poplar (Pt), apple (Md), *M. truncatula* (Mt), cucumber (Cs), and strawberry (Fv) pseudomolecules, recording separately the locations of genes with the highest and second-highest homology in the pseudomolecules (Additional file 4). In each comparison of a physical map with a pseudomolecule, we determined the beginning and end of a block of collinear genes, which we termed a synteny block (SB). We recorded cdBES that were collinear within a SB and used color coding to distinguish collinear genes from those that were not collinear (Additional file 4).

The total number of SBs detected ranged from 155 in the Jr-Fv comparison to 293 in the Jr-Pt comparison (Table 3). The number of SBs per pseudomolecule was the highest in the comparisons involving Mt, Cs, and Fv, the species with the smallest numbers of chromosomes (Table 3).

**Table 3:** Quantification of synteny involving 15,203 cdBES on the physical maps of the 16 *Juglans regia* chromosomes and the pseudomolecules of *Vitis vinifera* (Vv), *Populus trichocarpa* (Pt), *Malus domestica* (Md)*, Medicago truncatula* (Mt)*, Cucumis sativus* (Cs), and *Fragaria vesca* (Fv)

Measure	Vv	Pt	Md	Mt	Cs	Fv
Chromosome number	19	19	17	8	7	7
Number of syntenic blocks	227	293	211	180	185	155
No. of syntenic bocks per pseudomolecule	11.9a^a^	15.4a	12.4a	22.5b	26.4b	22.1b
Percent of collinear cdBES	30.2a	34.7a	19.0c	17.1cd	24.0b	13.7d
Number of collinear cdBES per syntenic block	21.7a	20.1ab	17.4b	16.4b	17.3b	19.3ab

^a^Means in rows followed by the same letters are not statistically different at the 5 % probability level

The mean number of collinear cdBES per SB was 21.7 and 20.1 in the Jr-Vv and Jr-Pt comparisons, respectively. In three other genome comparisons, Jr-Md, Jr-Mt, and Jr-Cs, the mean numbers of collinear genes per SB were smaller, ranging from 16.4 to 17.4 (Table 3), indicating that SBs were shorter and contained fewer collinear genes in the Jr-Md, Jr-Mt, and Jr-Cs genome comparisons than in the Jr-Vv and Jr-Pt comparisons. The comparison involving Fv was not significantly different from either group.

The most revealing was the percentage of the collinear cdBES of the total number investigated in a genome comparison. In the Jr-Pt and Jr-Vv comparisons, 34.7 and 30.2 % of all homologous genes detected in the Pt and Vv genomes were in collinear positions, respectively (Table 3). In the remaining four comparisons, the percentages of collinear genes were significantly lower: 19.0, 17.1, 24.0, and 13.7 % of the cdBES in the Jr-Md, Jr-Mt, Jr-Cs, and Jr-Fv comparisons, respectively.

**Fig. 2:**
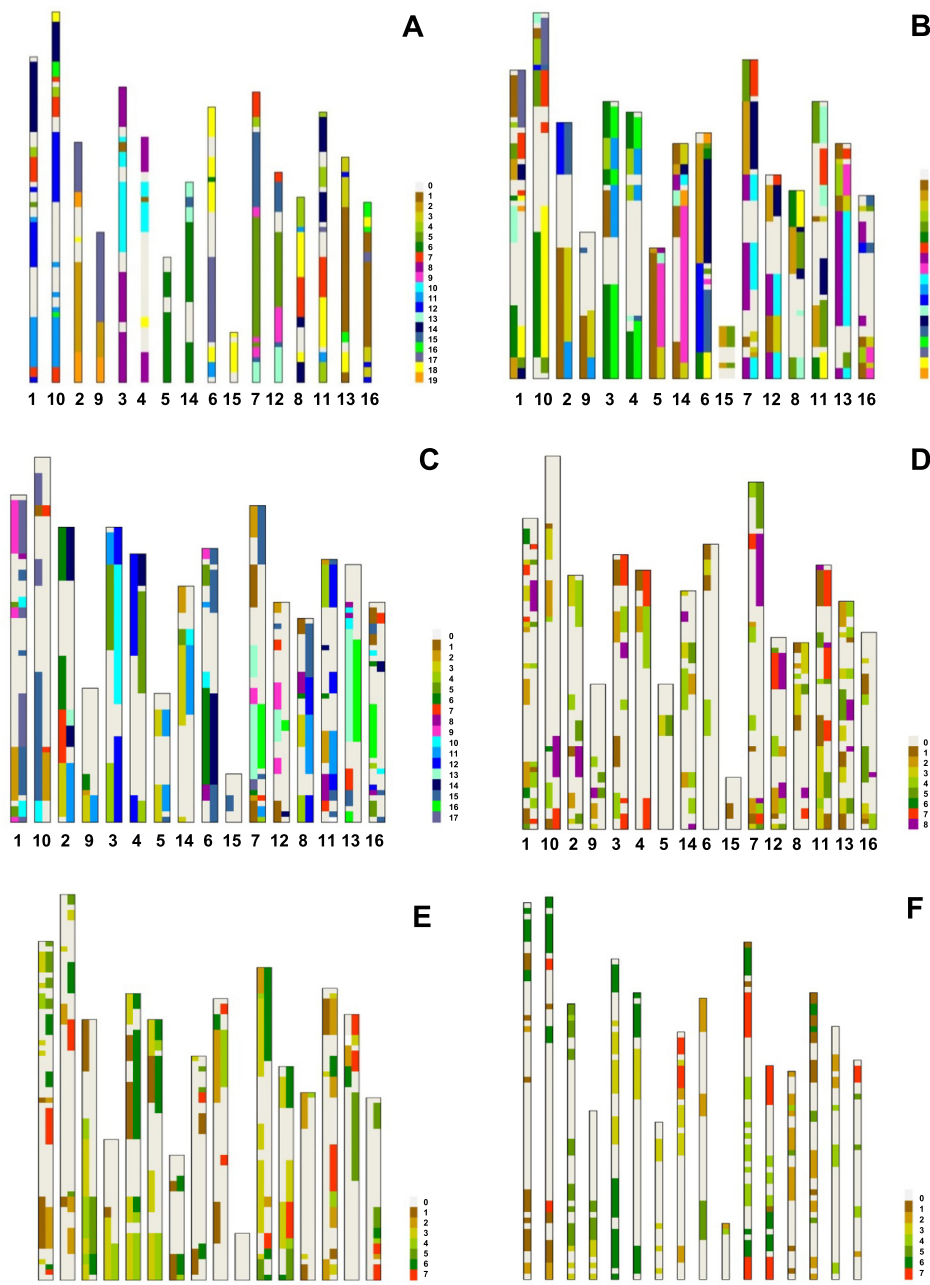
Synteny of walnut physical maps with the grape (**a**), poplar (**b**), apple (**c**), *Medicago truncatula* (**d**), cucumber (**e**), and strawberry (**f**) pseudomolecules. The 16 walnut physical maps are arranged as 8 pairs of homoeologues. Each physical map starts (0.0 Mb) at the top. The color code for pseudomolecules is shown at the right side of each panel. The regions devoid of synteny are white and labeled as a 0 pseudomolecule in the color code. Only the primary SBs are shown in the Jr-Vv (**a**) and Jr-Fv (**f**) genome comparison for the sake of clarity. In the remaining comparisons both the primary and secondary SBs (for definition see Methods) are shown as two tracks. Their placement into the left or right track is arbitrary

We illustrate these quantitative differences between the two groups of genome comparisons graphically in Fig. 2. SBs are shorter and more fragmented and regions devoid of synteny are longer in the Jr-Mt, Jr-Cs, and Jr-Fv comparisons than in the Jr-Vv and Jr-Pt comparisons. SBs in the Jr-Md comparison are intermediate between the two groups.

### Locations of walnut centromeres

The comparison of the Jr physical maps with the Vv, Pt, Md, Mt*,* Cs, and Fv pseudomolecules revealed the existence of a region in each physical map that was devoid of synteny across all six genome comparisons. Without exception, these gaps in synteny coincided with global minima in recombination rates (for Jr1 shown in Fig. 1 and for all chromosomes except for Jr15 in Additional file 5). We suggest that these are centromeric regions of the Jr chromosomes (Table 1).

### WGD

**Fig. 3:**
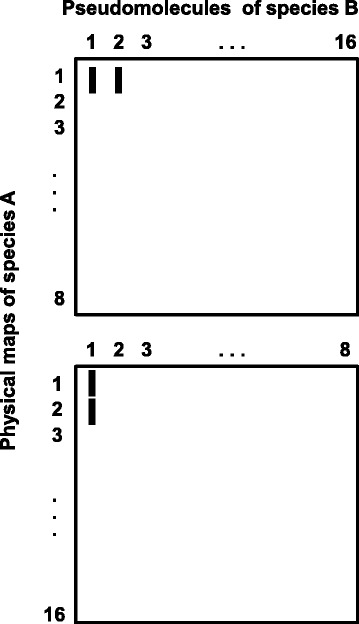
Outcomes of synteny analysis involving species with a WGD. Synteny is analyzed between hypothetical species A and B with a basic chromosome number of *x* = 8. The physical maps of species A and pseudomolecules of species B are arranged as in Table S3 in Additional file 4. Each vertical bar is a SB of homologous genes collinear in the genomes of species A and B. If a WGD has taken place in species B but not in species A, there are two pseudomolecules (1 and 2) of species B that are syntenic with a single physical map (1) of species A (upper panel). If a WGD has taken place in species A but not in species B, there are two physical maps of species A (1 and 2) that are syntenic with the same pseudomolecule (1) in species B (lower panel)

The number of SBs is expected to double in a genome comparison if a WGD has taken place in one of the compared genomes. If compared genomes are arranged as they are in the Excel Table S3 in Additional file 4, the cdBES arranged sequentially along the walnut physical maps from Jr1 to Jr16 on the vertical axis and pseudomolecules arranged one by one on the horizontal axis, a single walnut physical map is expected to be simultaneously syntenic with two pseudomolecules or their sections in the Vv, Pt, Md, Mt*,* Cs, and Fv genomes if any of these genomes harbors a WGD. As illustrated in the upper panel in Fig. 3, such a WGD manifests itself graphically as parallel SBs. If a WGD has taken place during evolution of the walnut genome, two different walnut physical maps are expected to be syntenic with the same pseudomolecule or its portion in each of the Vv, Pt, Md, Mt*,* Cs, and Fv genomes. As illustrated in the lower panel in Fig. 3, such a WGD manifests itself graphically as the same SB being duplicated in two different Jr physical maps. We encountered both patterns.

We sometimes observed two and in some cases three parallel SBs in the genome comparisons and arbitrarily named the longest SB as primary, shorter as secondary, and the shortest as tertiary. We measured the lengths of SBs in terms of the walnut physical map lengths in all genome comparisons and hence the lengths of SBs were comparable across all genome comparisons. The total length of the 16 walnut physical maps was 736.070 Mb (Table 1). The total length of the Jr-Vv primary SBs was 600.144 Mb (81.5 % of the total physical map length), that of the secondary SBs was 106.351 Mb (14.4 % of the total physical map length), and that of tertiary SBs was 6.920 Mb (0.9 % of the total physical map length) (Table 4 and Additional file 4). The existence of three parallel SBs in the Jr-Vv genome comparison was consistent with the γ triplication in the Vv genome lineage [26, 40].

**Table 4:** The lengths of the primary, secondary, and tertiary SBs in the comparison of 16 *Juglans regia* (Jr) chromosomes (total physical map length = 736070 Mb) with the pseudomolecules of *Vitis vinifera* (Vv), *Populus trichocarpa* (Pt), *Malus domestica* (Md)*, Medicago truncatula* (Mt)*, Cucumis sativus* (Cs), and *Fragaria vesca* (Fv)

Measure	Jr-Vv	Jr-Pt	Jr-Md	Jr-Mt	Jr-Cs	Jr-Fv
Chromosome number	19	19	17	8	7	7
Total length of the primary SBs (Mb)	600.144a^a^	556.891a	535.223a	349.414c	459.251b	298.298c
Percent of the Jr physical map	82	76	73	49	62	41
Total length of the secondary SBs (Mb)	106.351b^a^	379.983a	267.060a	133.824b	178.461b	2.871d
Percent of the Jr physical map	14	52	36	19	22	1
Length of the tertiary SBs (Mb)	6.920^b^	62.581	6.692	4.715	14.436	0
Percent of the *Jr* physical map	1	9	1	1	2	0
Ratio of secondary SBs to primary SBs	0.17	0.68	0.50	0.39	0.39	0.01

^a^Means in rows followed by the same letter were not significantly different at the 5 % probability level. ^b^Statistical significance of differences between means was not tested because of large numbers of zeroes

For reasons pointed out earlier we used the Jr-Vv comparison as a baseline reference in search for WGDs in the remaining genomes. The total length of the primary SBs in the Jr-Pt and Jr-Md genome comparisons did not significantly differ from that in the Jr-Vv comparison but was significantly shorter in the Jr-Mt, Jr-Cs, and Jr-Fv genome comparisons (Table 4). Like the percentage of collinear genes earlier, this measure showed that synteny of the Jr genome with genomes of herbs, Cs, Mt, and Fv, was more eroded than synteny of the Jr genome with the genomes of woody perennials, Vv, Pt, and Md (Table 4). The secondary SBs were significantly shorter in the Jr-Vv, Jr-Mt, Jr-Cs, and Jr-Fv genome comparisons than in the Jr-Pt and Jr-Md genome comparisons (Table 4). Except for the Jr-Vv genome comparison, the absolute lengths of the primary and secondary SBs were related within individual genome comparisons and depended on the overall level of synteny erosion. We therefore used the ratio of secondary to primary (S/P) SB lengths as measure of SB duplication. We used the S/P SB ratio in the Jr-Vv genome comparison as a benchmark to test for the presence of a WGD in other genome comparisons.

The S/P SB ratio was 0.17 in the Jr-Vv comparison but 0.68, 0.50, 0.39 and 0.39 in the Jr-Pt, Jr-Md, Jr-Mt, and Jr-Cs comparisons, respectively (Table 4). Thus, relative to the length of the primary SBs the lengths of the secondary SBs were longer in the Jr-Pt, Jr-Md, Jr-Mt, and Jr-Cs comparisons than in the Jr-Vv comparison. We therefore concluded that, in addition to the *γ* triplication, which is presumably present in these four lineages, a more recent WGD occurred in each of these lineages. The Jr-Fv comparison was exceptional since only two secondary and no tertiary SBs were detected. The S/P SB ratio was 0.01 reflecting the extremely short length of the secondary SBs in the Fv genome and indicating that no recent WGD occurred in the Fv lineage.

In each genome comparison, we recorded the most similar and the second-most similar homologous gene to a walnut cdBES separately (Additional file 4). In duplicated regions, the two types of homologous genes alternated along the primary and secondary SBs in each of the Jr-Pt, Jr-Md, Jr-Mt, and Jr-Cs genome comparisons (the Jr-Fv comparison was not considered because of the near absence of secondary SBs), which indicated that the primary and secondary SBs were overall equidistant to the corresponding section of the Jr physical map. Furthermore, these patterns were dissimilar among the comparisons (Additional file 4), which indicated that the WGDs in the Pt, Md, Mt, and Cs lineages occurred after the lineages had diverged from the Jr lineage and each was an independent WGD event.

We used the following procedure to determine whether a WGD occurred in the Jr lineage. For each SB identified in the Jr-Vv genome comparison, we determined if there was a duplicated SB on another walnut chromosome, as illustrated in the bottom panel of Fig. 3. To quantify the length of the duplication, we counted the numbers of collinear cdBES in the duplicated SBs. Of 3,742 collinear cdBES in the Jr-Vv comparison, 77.3 % were located in SBs duplicated on different Jr chromosomes. We performed a similar analysis using SBs based on the Jr-Pt genome comparison. In that analysis, 72.2 % of the 3,543 collinear cdBES were located in SBs duplicated on different Jr chromosomes. Both sets of data suggested that a WGD occurred in the Jr lineage.

For 15 of the 16 Jr chromosomes, we always found one other Jr chromosome that contained most of the duplicated SBs (Additional file 6). We suggest that these pairs of chromosomes are ancient homoeologues. These homoeologous relationships were reciprocal. For example, when Jr1 was used as a query, the highest percentage (49.7 %) of collinear cdBESs in the Jr-Vv comparison was located in the SBs duplicated in Jr10 (Additional file 6). When Jr10 was used as a query, the highest percentage (60.4 %) of collinear cdBESs was located in SBs duplicated in Jr1. Except for a single pair (Jr6-Jr15), the same strong reciprocal relationships were observed in other homoeologous chromosome pairs (Additional file 6). In the Jr6-Jr15 pair the relationships were not reciprocal. When Jr15 was used as a query, the corresponding chromosome was Jr6 using both the Jr-Vv and Jr-Pt SBs. However, when Jr6 was used as a query, other chromosomes contained greater numbers of collinear cdBES located in duplicated SBs other than Jr15. We suggest that this lack of reciprocity was caused by the short length of chromosome Jr15 (Fig. 2). With the sole exception of this chromosome pair, the remaining walnut chromosomes showed clearly defined homoeologous relationships (Table 5).

**Table 5:** Pairs of walnut homoeologous chromosomes and predicted structure of the eight ancestral chromosomes in terms of Jr-Vv SBs

Walnut homoeologues		Ancestral chromosome structure^a^
Jr1	Jr10	Vv7, Vv11, (Vv5)^b^, Vv12, Vv7,Vv 4, (Vv16), Vv14, (Vv18)
Jr2	Jr9	Vv17, Vv19, Vv2
Jr3	Jr4	Vv8, Vv10, Vv1, Vv10, Vv8
Jr5	Jr14	Vv6, (Vv15), (Vv13), (Vv15)
Jr6	Jr15	Vv18, (Vv17)
Jr7	Jr12	Vv7, (Vv4), Vv15, Vv5, Vv9, Vv15, Vv13
Jr8	Jr11	Vv4, Vv11, Vv18, Vv7, Vv14
Jr13	Jr16	Vv16, Vv1, Vv3, Vv12

^a^The tips of the short arms are to the left

^b^SBs in parentheses were present in only one of the two Jr homoeologues but were not detected anywhere else in the genome, and we therefore assumed to be deleted from the other homoeologue

The presence of duplicated SBs in homoeologous chromosomes is evident in Fig. 2, in which the order of the Jr chromosomes was arranged so that the homoeologous chromosomes are side-by-side. The homoeologous relationships are the most clearly apparent in the Jr-Vv genome comparison. The same primary SBs and in the same order are usually conserved within homoeologous chromosome pairs, although chromosomes Jr11 and Jr16 have to be inverted to be parallel to their homoeologues, Jr8 and Jr13, respectively. While the order of SBs tends to be conserved, the lengths of corresponding SBs relative to each other vary greatly in homoeologous pairs (Fig. 2).

**Fig. 4:**
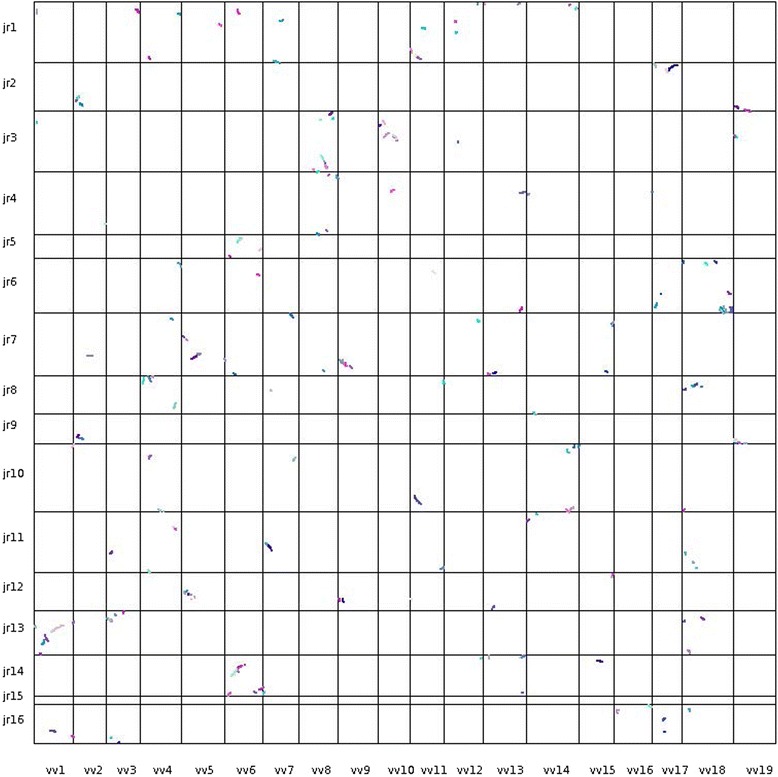
A dot plot of the 16 walnut physical maps on the vertical axis, with the starting nucleotide of each physical map at the top, and the 19 grape pseudomolecules on the horizontal axis, with the staring nucleotide on the left. Each dot represents a collinear gene pair identified by MCScanX between walnut and grape. Different colors of dots represent different collinear blocks of genes

**Fig. 5:**
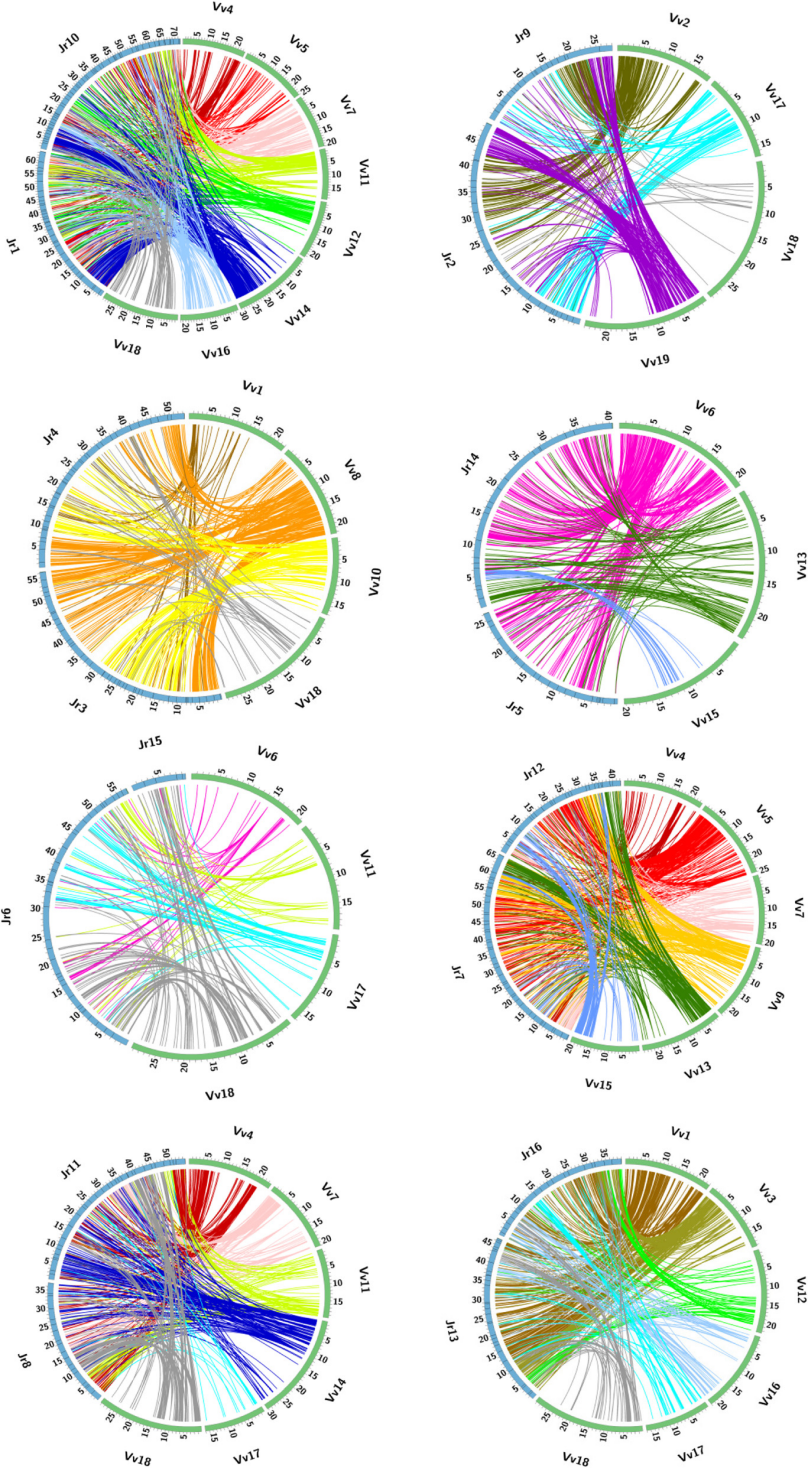
Duplications of SBs in the putative walnut homoeologous chromosome pairs. Jr homoeologous chromosomes are depicted by blue segments of the circle. Only grape pseudomolecules (green sections of the circles) that are partially syntenic with the indicated walnut chromosomes are shown. The scales on the pseudomolecules and physical maps are in Mb. Colored lines connect cdBES on Jr physical maps with homologous genes on Vv pseudomolecules. Note that bundles of lines emanating from a single Vv pseudomolecule or a portion of it bifurcate and lead to both Jr homoeologous chromosomes, indicating duplicated SBs between walnut homoeologues

We also constructed a global dot plot of Jr physical maps with the Vv pseudomolcules (Fig. 4) and circular plots of each putative Jr homoeologous chromosome pair with the pseudomolecules of relevant Vv chromosomes (Fig. 5) to illustrate the WGD in the walnut genome. The dot plot confirms most of the homoeologous relationships inferred in Additional file 6 and shown in Fig. 2a as well as relationships between Jr and Vv chromosomes summarized in Fig. 2a. Nevertheless, the great fragmentation and diffused nature of SBs makes the dot plot of limited value for revealing intra-genomic synteny in the walnut genome. The circular graphs (Fig. 5) illustrate the relationships more clearly than the dot plot and capture some of the duplicated SBs lost in data filtering during the construction of the dot plot. Bundles of lines connecting homologous Jr and Vv genes and emanating from Vv pseudomolecules usually bifurcate and clearly show that SBs are duplicated in the Jr genome. They also show that the sequence of duplicated SBs is often similar in putative Jr homoeologues. We used the information we had on sharing of Jr-Vv SBs by the Jr homoeologous chromosome pairs to suggest the composition of the eight chromosomes in the genome of the diploid ancestor of Jr.

### *K*s estimation

**Fig. 6:**
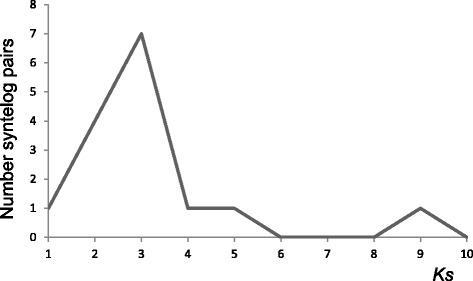
Frequencies of *K*s values among 15 pairs of walnut syntelogs

To assess the rate of nucleotide substitution at silent sites in the walnut lineage we searched for syntelogs in the walnut genome using the following criteria: cdBES had to contain exonic sequences, be present in duplicated SBs in the walnut genome, and both be collinear with a homologous gene in the Vv or Pt genome. We analyzed 62 syntelogs but only in 15 were the same exons present in the BES. We computed *K*s for each of the 15 pairs and plotted the values. Based on the plot (Fig. 6) we eliminated a single high outlying value. None of the remaining 14 genes were annotated as a transposable element (Additional file 7). Using these 14 gene pairs we obtained *K*s = 0.27429 ± 0.0876 nucleotide substitution per silent site (mean and standard deviation, respectively).

## Discussion

### Recombination rates and the maps

The average length of the walnut LGs was 65.6 cM, and all LGs were short, ranging from 37 to 97.3 cM, suggesting that a short LG length was a global property of the walnut genome. The genetic map was built from 1,525 SNP markers, and it is therefore unlikely that insufficient marker coverage was the cause of short LGs. The map was *de facto* a female backcross map, and it is possible that a low recombination rate in the female was the cause of short LGs, since sex-related differences in map lengths are common in plants [46–53].

Short LGs have been reported in other woody perennials, such as the apple and pear [54–56], the grape [57, 58], and the oak, *Quercus robur,* [59, 60]. It is therefore also possible that low recombination rates are part of the reproductive strategy of woody perennials. Pericentromeric regions of Jr chromosomes showed low recombination rates and most of the recombination took place in the subterminal regions of the chromosomes. Distal localization of recombination and overall short genetic lengths of chromosomes are adaptations attributed to high levels of outcrossing [61–63].

It is also possible that the low recombination rates in the walnut, apple, pear, grape, and oak genomes are an adjustment of recombination to the high numbers of chromosomes in those genomes. The walnut genetic map was produced with a similar number of genetic markers as the genetic map of *Aegilops tauschii,* an inbreeding grass species with a relatively high level of recombination per chromosome but with only 7 chromosomes. The total length of the 16 walnut LGs was 1,049.5 cM and the total length of the 7 *Ae. tauschii* LGs was 10 % longer, at 1,166.8 cM [15]. The large number of chromosomes in the walnut genome offsets the low recombination in individual walnut chromosomes.

We deployed in this study only SNPs discovered in Chandler, which increased the likelihood of BAC anchoring on the genetic map and facilitated the construction of the physical map. The variety ‘Payne’ is a shared ancestor of both parents of Chandler. Neglecting the potential inbreeding of Payne, the Chandler inbreeding coefficient *F* was estimated as 0.0625. Hence, at least 6.25 % of the Chandler genes are expected to be autozygous, and autozygous gene blocks would be devoid of SNPs. Such regions of the genome could not be genetically mapped and would appear as gaps on the genetic map. They would not affect the length of the genetic map if they were located interstitially because crossovers taking place in homozygous regions result in recombination of heterozygous flanking markers. Gaps located terminally may however escape detection because there is no flanking marker on the distal end of a gap to reflect crossovers in the homozygous region. A terminally located autozygous region will therefore reduce the genetic map length, and could be a factor shortening the lengths of specific walnut LGs. This possibility is relevant to the very short LG15.

To assess empirically the seriousness of autozygosity for the walnut genetic map, we again compared it with the *Ae. tauschii* genetic map. The *Ae. tauschii* mapping population was produced by crossing parents from different subspecies and there was little chance for autozygosity in that population [15]. Each *Ae. tauschii* gap was divided by 2.5 to take into account longer *Ae. tauschii* LGs. The longest gap on the *Ae. tauschii* map scaled to be comparable to the gaps on the walnut map equaled to 8.0 cM. There were ten gaps longer than 8.0 cM on the walnut genetic map (Additional file 8), indicating that gaps were indeed longer on the walnut genetic map than on a map based on a population in which autozygosity was not a factor. The total length of the 10 gaps was 129.15 cM (12.3 % of the total genetic length of the walnut map). Because these gaps were in regions with high recombination rates, they were physically relatively short, totaling 23.6 Mb (3.2 % of the physical map length). The combined effects of the interstitial and terminal gaps on the utility of the physical map were therefore probably minor. This is also suggested by the fact that the estimated total length of the physical map anchored on the genetic map, about 736 Mb, was close to the estimated size of the walnut genome, 606 Mb (Horjales et al. 2003 in http://data.kew.org/cvalues/).

### WGD

Whole genome duplications, in addition to the *γ* triplication detected in Vv [26], have been previously reported in the Pt [8], Md [29], Mt [64], and Cs [31] genomes. We confirmed each of these events and uncovered a WGD in the Jr genome. We assigned the 16 Jr chromosomes into 8 pairs of putative homoeologues, which, except for the Jr6-Jr15 pair, usually showed the same SBs often arranged in the same order. It was suggested that the *γ* triplication generated a genome with *n* = 21 [65]. The eight homoeologous chromosome pairs in the haploid Jr genome suggests that *n* = 16 did not evolve from *n* = 21 by dysploid reduction but from *n =* 8 by WGD. Except for genera *Cyclocarya* and *Platycarya*, the rest of Juglandaceae share *n* = 16 [66]. In *Cyclocarya, n* = 28 is probably another round of WGD. Although different counts, *n* = 14, 12, and 11, have been reported for *Platycaya strobilacea* [67–69], the fact that all of them are lower than *n* = 16 suggests that the actual chromosome number is less than 16. The presence of *n* = 16 in both subfamilies of Juglandaceae, Juglandoidae and Engelhardioidae [66], leaves little doubt that *n* = 16 is the ancestral state in Juglandaceae and the chromosome number in *Platycarya strobilacea* was derived from *n* = 16 by dysploid reduction.

The most parsimonious model of chromosome number evolution in Juglandaceae is the WGD preceding the radiation of the Juglandaceae. Otherwise, we would have to assume that a WGD happened independently in each of the lineages within the family and was always accompanied by the extinction of the diploid ancestor, which is unlikely. Our hypothesis that the ancestral state was *x* = 8 is supported by the presence of *n* = 8 in the monotypic genus *Roiptelea* [70], which contains a single species *R. chiliantha. Roiptelea* has been considered a monotypic family of Fagales and the sister clade of Juglandaceae [71]. It was recently transferred into the family Juglandaceae (APG III system), reflecting the close affinity to Juglandaceae. Chromosome number *n* = 8 is also present in Myricaceae [72], which also is closely related to Juglandaceae [73]. Since the fossil record places the radiation of Juglandaceae to the Paleocene [74], 56 to 66 MYA, the WGD very likely happened in this time window. We propose to name this WGD as the “juglandoid” WGD.

Timing the juglandoid WGD to about 60 MYA adds one more WGD to a growing number of WGDs that have occurred near the Cretaceous-Tertiary (K-T) boundary, about 66 MYA [75, 76]. The clustering of WGD events at the K-T boundary has been attributed to a greater ability of polyploids to survive the adverse environmental conditions and mass extinction associated with the K-T boundary [75], although, as pointed out [76, 77], other factors may have played a role.

### Polyploidy-dysploidy (P-D) cycles

The juglandoid WGD duplicated an ancestral genome with *x* = 8 into a genome with *n* = 16. In the following 60 MY, the size of Jr15 has been reduced. In the Jr karyotype, Jr15 is a small telocentric chromosome [78]. The analysis of its homoeologue, Jr6, suggested that a large portion (48 to 64 %) of Jr15 may have been deleted. An actual dysploid reduction has taken place in *Platycarya strobilacea,* from *n* = 16 to either *n* = 14 or *n* = 12.

Similar dysploid reductions have taken place in the poplar and apple lineages. In the former lineage, the salicoid WGD that originated about 65 MYA duplicated a genome with *x* = 10 and produced a genome with n = 20. This number of chromosomes was reduced to *n* = 19 [8], which is widespread in Salicaceae (IPCN, http://www.tropicos.org/Project/IPCN), in which poplar is classified. *Malus* is a member of tribe Pyreae of the Rosaceae. Pyreae have uniformly *n* = 17. This chromosome number originated by a WGD which doubled a genome with *x* = 9. The resulting *n* = 18 was reduced by dysploidy to *n* = 17 [29]. Strawberry *x* = 7 very likely evolved from x = 9 [30].

In each of these P-D cycles, the dysploid phase resulted in a reduction in chromosome number, never in an increase. A similar trend exists in grasses. In the lineages in which the availability of genomic data allowed thorough analysis of P-D cycles, the dysploid phase always resulted in a reduction in chromosome number, never in its increase [15, 17, 32, 79, 80]. We cannot offer an explanation of this tendency except for suggesting that processes increasing the numbers of bibrachial chromosomes are inherently more complex, and therefore less likely to take place, than those reducing them [61].

The rates of dysploid reduction in the walnut, poplar, and apple P-D cycles are puzzling. If the ancestral genome of Rosids indeed had *n* = 21 as suggested by Salse [65], the rate of dysploid reduction had to be precipitous in the 60 MY preceding the WGDs in the walnut, apple, and poplar lineages, which were timed to 60 (our data), 60 [29], and 65 [8] MYA, respectively, to generate *x* = 8, 9, and 10 that preceded the WGDs in these lineages, respectively. In the 60 MY following the WGDs, either none or only one or few chromosomes were eliminated by dysploidy in each lineage. We can offer no explanation of this glaring discrepancy in the rates of dysploid reduction.

### Nucleotide substitution rates

We concluded that the juglandoid WGD preceded or occurred at the time of the radiation of Juglandaceae, which based on the fossil record radiated in the Paleoceae [74], 56 to 66 MYA. Using a midpoint of 60 MYA and our estimate of *K*s = 0.27429, we obtained the nucleotide substitution rate *r* = 2.29 × 10^−9^ nucleotide year^−1^ for the walnut lineage. This rate is 6.5 times slower than *r* = 1.5 × 10^−8^ nucleotide year^−1^ reported for the *Arabidopsis* lineage [81] and 4.7 times slower than *r* = 1.08 × 10^−8^ nucleotide year^−1^ reported for the Mt lineage [64] but is very similar to the rates inferred for palms, 2.61 × 10^−9^ nucleotide year^−1^ [5] and a similar rate for the poplar [8]. The fact that our estimate of *r* for woody perennials in Fagales is close to those of other woody perennials is consistent with the hypothesis that the molecular clock in plants is related to the life-cycle length [11] and ticks more slowly in long-lived woody perennials than in short-lived herbs.

### Synteny erosion rate

Synteny of the Jr genome with genomes of short-lived herbs, Mt, Cs, and Fv, was more eroded than synteny of the Jr genome with the genomes of long-lived woody perennials, Vv and Pt. We did not include *A. thaliana* in our study*,* because synteny of its genome with that of the grape genome has already been shown to be more eroded than synteny between the genomes of the grape and poplar, two woody perennials [26]. Synteny of Jr with the Md genome was intermediate between the two groups, although Md is a woody perennial. Rosaceae are a mixture of long-lived woody perennials and short-lived herbs and it is possible that the apple evolutionary lineage included or was preceded by species with intermediate life-cycles. Even in Rosaceae, however, the relationship between the life-cycle length and synteny erosion holds since synteny of Fv, an herb, with Jr was more eroded than that of Md, long-lived woody perennial. While 19 % of cdBES were collinear in the Jr-Md genome comparison, only 13.7 % were collinear in the Jr-Fv genome comparison. The greater erosion of synteny in the Fv than in Md genome may account for the failure to detect the γ triplication in the Fv genome (our data and those in [30]) and its detection in the more slowly evolving apple genome [29]. While in the Jr-Vv and Jr-Pt comparisons 30.2 and 34.7 % genes were in collinear locations, only 19.0, 17.1, 24.0, and 13.7 % genes were in collinear locations in the Jr-Md, Jr-Mt, Jr-Cs, and Jr-Fv genome comparisons, although phylogenetically the Md, Mt, Cs, and Fv genomes are more closely related to the Jr genome than to the Vv and Pt genomes [27, 36–38, 82].

Is it possible that some other factor rather than the life-cycle length was responsible for the differences in synteny erosion with the walnut genome among the six investigated genomes? In theory, a WGD could accelerate synteny erosion by reducing the strength of purifying selection acting on individual genes. Recent WGDs took place in the Pt, Md, Mt, and Cs lineages but not in the Vv and Fv lineages. Yet, the rates of synteny erosion in the Vv and Fv lineages greatly differ. Additionally, genome erosion is faster in the Fv lineage, which is devoid of WGD, than in the Md lineage, in which a WGD took place. Thus, WGD do not seem to have strong effects on the rate of synteny erosion. It is possible that the rate of synteny erosion and the rate of molecular clock are indirectly related to life-cycle length and more directly related to the average life expectancy or some other demographic or developmental factor that is correlated with life-cycle length in angiosperms.

## Conclusion

Compared to short-lived herbs, long-lived woody perennials show slow rates of nucleotide substitution and slow rates of synteny erosion. Slow rates of molecular clock and slow rates of synteny erosion in long-lived angiosperms may be manifested in other facets of genome divergence. Chromosome pairing and recombination in interspecific hybrids is one example [83]. Reproductive isolation between species, which of all angiosperm life-forms is the weakest between woody perennials [84], is another.

## Methods

### Mapping population

We generated the Chandler x Idaho population by controlled pollination of Chandler with Idaho pollen. Chandler is a product of the University of California-Davis walnut breeding program. Idaho is a tree of unknown parentage originally identified in Parma, Idaho. We germinated 425 Chandler x Idaho F_1_ nuts in the greenhouse, transplanted the saplings to a commercial nursery for one year, and then to an orchard at UC Davis.

### Markers and the construction of the genetic map

We constructed the genetic map with the walnut 6 K Infinium SNP assay [42]. SNPs were designated according to the BES of their origin. H and M stand for *Hin*dIII and *Mbo*I, libraries, respectively, which is followed by the three digit number of the 384 plate in the BAC library, followed by the row and column designations. Since all BES were generated by using the reverse sequencing primer, all marker names end with “r”. Only a single BES was developed per BAC clone and therefore only a single SNP marker can be present in a BAC clone. Genomic DNA of the 425 F_1_ individuals and the parents were genotyped with the Infinium assay following the Illumina Inc. (San Diego, USA) protocols at the UC Davis Genome Center. We analyzed data with GenomeStudio v. 1.0 (Illumina) using the Genotyping Module set for a GenCall threshold of 0.15. The software automatically determines the cluster positions of the three codominant monohybrid genotypes for each SNP and displays them in normalized graphs. We set GenTrain score ≥0.50, minor allele frequency ≥0.15, and call rate >80 % to filter out low quality SNPs and manually removed SNPs for which clustering of samples was distorted, had one of the parental DNAs missing, or had > 25 % of the individuals not called in clusters. Because SNPs were discovered within Chandler, we used in the linkage analysis only markers that segregated in Chandler. To identify markers showing segregation distortion, we performed a Chi-square test at *P* = 0.01 to identify markers that significantly differed from the 1:1 segregation ratio. We retained the final set of 1,706 markers heterozygous in Chandler and homozygous in Idaho for mapping, using JoinMap v3.0 and v4.0 software. We set LOD score at 5 and used the Kosambi function for map distance calculation. We drew the maps with MapChart v2.2 for Windows.

### BAC fingerprinting, fingerprint editing, and BAC contig assembly

We fingerprinted 124,890 BAC clones, 62,954 from the *Mbo*I and 61,939 from the *Hin*dIII libraries [41] with a SNaPshot high-information content fingerprint (HICF) method [43] modified by Gu et al. [44]. We size-fractionated the restriction fragments on an ABI3730XL DNA analyzer using LIZ 1200 size standard (Applied Biosystems, Foster City, California) and the GeneMapper software.

Outputs of size-calling files were automatically edited with the FP Miner program as described elsewhere [44]. The program distinguished peaks corresponding to restriction fragments from peaks generated by background and removed vector restriction fragments from the profiles. The program also removed sub-standard profiles and cross-contaminated profiles that could negatively affect BAC contig assembly. We considered clones to be cross-contaminated if they resided in neighboring wells and shared 30 % or more of the mean number of fragments in their profiles. We used files generated by FP Miner for contig assembly with the FPC.

We assembled contigs with FPC software v9.3 (http://www.agcol.arizona.edu/software/fpc/r94.html), using fragments within the 70 to 1,000 bp range. We performed the initial assembly with tolerance of 5 (=0.5 bp) and Sulston score cutoff 1 × 10^−65^. Following the initial assembly, we performed several rounds of DQer until no contig contained 15 % or more Q-clones. Then we performed several rounds of end-to-end merging and single-to-end merging at progressively lower cutoff stringencies. We set the “Best of” parameter to 100 builds.

### Physical map construction

We manually edited all contigs containing markers and also those that did not contain markers but were ≥ 1 Mb long, following a procedure described earlier [17]. We anchored contigs by searching BES for nucleotide sequences of SNP markers on the genetic map, using FPC module “Merging Markers” followed by manual examination of the locations of markers integrated into each BAC contig. We deemed the anchoring of a contig validated if all markers in the contig were in a single region of the genetic map. If they were in two separate regions, we examined the CB (consensus band) map of the contig using the CB map FPC routine, and identified the false join. We then manually disjoined the chimeric contig using FPC tools. For each contig devoid of markers and ≥ 1 Mb long, we examined its CB map for the presence of chimeric clones and disjoined chimeric contigs.

### Synteny analyses and quantification

We vertically arranged walnut cdBES based on their order within BAC contigs and the order of BAC contigs along the genetic map and determined homology of each cdBES with the grape, v.145, poplar, v.210, apple, v.196, *Medicago truncatula,* v.198, cucumber, v.122, and strawberry, v.226 non-redundant protein databases (http://phytozome.jgi.doe.gov/) using BLASTX and *P* < 1 E-5. We placed the starting nucleotides of homologous genes with the lowest and second-lowest *P* values at the intersection of the cdBES row and the appropriate pseudomolecule column. We also recorded whether or not the gene was in a collinear position relative to surrounding genes. We assumed collinearity if the starting nucleotide of the homologous gene was consistent with the regular increase or decrease of starting nucleotides of surrounding genes along a pseudomolecule. We called a group of collinear genes an SB if at least three different collinear genes were present in a group of homologous genes and the SB was about 0.5 Mb or more in terms of the walnut minimum tiling path length (MTP).

We used the following strategy for synteny quantification. Because of WGDs in compared genomes, there could be more than one SB detected between walnut and a compared genome and these would appear as parallel SBs in the synteny table (Additional file 4). Let the symbol SB_*ij*_ stand for the *i*^th^ SB (*i* = 1, 2,… n) and *j*^*th*^ parallel version of it (*j* = 1, 2, or 3). A region of the walnut physical map was syntenic with either none, one (SB_i1_), two (SB_i1,_ + SB_i2_), or three (SB_i1_ + SB_i2_ + SB_i3_) parallel SBs in a compared genome. We defined ΣSB_i1_ as the length of the primary SBs, ΣSB_i2_ as the length of the secondary SBs, and ΣSB_i3_ as the length of the tertiary SBs. Parallel SBs were assigned into the three categories according to their lengths: the longest was SB_i1_ and the shortest was SB_i3_.

### Estimation of *K*s and nucleotide substitution rate

We aligned coding sequences of syntelogs with ClustalW and computed the number of substitutions per synonymous site *K*s with the MEGA software (v6.0) [85] using the Nei-Gojobori model of nucleotide evolution accounting for multiple substitutions per site [85]. We averaged the *K*s values for individual syntelogs and computed the rate of nucleotide substitution in silent codon positions as *r* = *K*s/2*t,* where *r* is the rate of nucleotide substitution per synonymous site year^−1^, *K*s is mean number of nucleotide substitutions per synonymous site, and *t* is time of syntelog divergence in years.

### Recombination rates along walnut MTP

We selected a BAC clone containing a mapped marker in its BES every 5 Mb along the physical map of a walnut chromosome. We divided the distance between the markers in cM by distance in Mb to compute the recombination rate (cM/Mb) in the interval and averaged the means of two neighboring intervals. The 5-Mb window was then moved into the next position by 5 Mb. In the last step, there was only one 5-Mb window, rather than two, and we could not compute the average of two 5-Mb windows. We therefore repeated the process moving in the opposite direction and computed the means of the two estimates. Mean recombination rates were plotted along the physical map of a chromosome.

### Circular graphs and dot plot

Circular graphs (Circos v0.67) [86] were employed to plot synteny between specific walnut physical maps and grape pseudomolecules. BLASTX was used to identify for each walnut cdBES a Vv homologous gene at cutoff *P* < 1E-5. Only top hit above the cutoff was used to link a gene pair in a graph. The dot plot between the walnut and grape genomes was constructed with MCScanX [87]. Four sets of BLAST outcome (walnut vs. grape, grape vs. walnut, walnut vs. walnut, and grape vs. grape) at cutoff P < 1E-5 and -m8 output option were merged as alignment input, GFF file in simple version for both grape and walnut coding genes were merged as coordination input, figures were plotted by using default parameters in the Linux environment.

### Statistical analyses

To determine statistical significance among means, we used data for the 16 individual walnut physical maps as variables and analyzed them with the GLM procedure in SAS v.9.3 for Windows. If we analyzed values ranging between 0.0 and 1.0 we transformed the data with arcsine prior to analysis of variance. Differences between means were analyzed with the LSD procedure at α = 0.05.

## Availability of supporting data

cdBES have the names of the BACs from which they originated. They are listed in column E (heading “BAC”) of the Excel Table S3 in Additional file 4. Their FASTA files are in the GenBank database (http://www.ncbi.nlm.nih.gov/nucgss/?term=Juglans+regia). The database of the fingerprinted BAC clones, BAC contigs and integrated SNP markers is available at http://phymap.ucdavis.edu/walnut/.

## Additional files

Additional file 1: Table S1. Excel spreadsheet listing 1,525 SNP markers and their locations on the genetic maps of the 16 walnut chromosomes. (XLSX 106 kb) Additional file 2: Figure S1. Graphical presentation of 16 walnut linkage groups. (PDF 1.09 mb) Additional file 3: Table S2. Excel spreadsheet listing all 1,031 BAC contigs including the nested BAC contigs that were removed from the physical map to generate a non-redundant physical map. (XLSX 57.6 kb) Additional file 4: Table S3. Excel file listing the locations of 15,203 cdBES on the physical map, their annotation, and synteny of the 16 Jr physical maps with the Vv, Pt, Md, Mt, Cs, and Fv pseudomolecules. The table shows the coordinates on the respective pseudomolecules for Vv, Pt, Md, Mt, Cs, and Fv genes with the lowest (in the columns designated .1) and second-lowest if obtained (in the columns designated .2) E-value. We colored cells with collinear genes with any color except for white or green. We used different colors to distinguish neighboring SBs from each other. We used white cell color to indicate non-collinear genes and green cell color to indicate collinear genes with the second-lowest E-value. (XLSX 7.26 mb) Additional file 5: Figure S2. Graphs of recombination rates across 15 of the 16 walnut physical maps. We indicate the location of synteny gap on each physical map as a thick horizontal bar. (PDF 311 kb) Additional file 6: Table S4. Numbers and percentages of walnut cdBES collinear with genes in the grape and poplar pseudomolecules located in SBs duplicated in the walnut genome. (DOCX 19.0 kb) Additional file 7: Table S5. Pairs of syntelogs and their annotations used for estimation of *K*s. (DOCX 16.3 kb) Additional file 8: Table S6. Characteristics and locations of gaps larger than 8 cM in the 16 walnut linkage groups. (DOCX 16.2 kb)

## Abbreviations

APG III system: Angiosperm phylogeny III system
BAC: Bacterial artificial chromosome
BES: BAC-end sequence
CB: Consensus band map program in FPC
cdBES: BES containing coding sequences
cM: centimorgan
Cs: *Cucumis sativus*
FPC: Name of package of programs for BAC contig assembly and manipulation
Fv: *Fragaria vesca*
Jr: *Juglans regia*
L50: The length of the smallest contig in a N50 set
Mb: Million base pairs
Md: *Malus domestica*
Mt: *Medicago truncatula*
MY: Million years
MYA: Million years ago
N50: The length for which the contigs of that length or longer contain at least half of the sum of the lengths
P-D: Polyploidy-dysploidy
Pt: *Populus trichocarpa*
SB: Synteny block
TE: Transposable element
Vv: *Vitis vinifera*
WGD: Whole genome duplication
