# Supplementary material for: Synteny analysis in Rosids with a walnut physical map reveals slow genome evolution in long-lived woody perennials
Source: BMC Genomics. 2015 Sep 17;16(1):707. doi: 10.1186/s12864-015-1906-5 (PMC4574618; doi:10.1186/s12864-015-1906-5)
Supplement: Additional file 5: Figure S2. — Graphs of recombination rates across 15 of the 16 walnut physical maps. We indicate the location of synteny gap on each physical map as a thick horizontal bar. (PDF 311 kb) [file 12864_2015_1906_MOESM5_ESM.pdf]

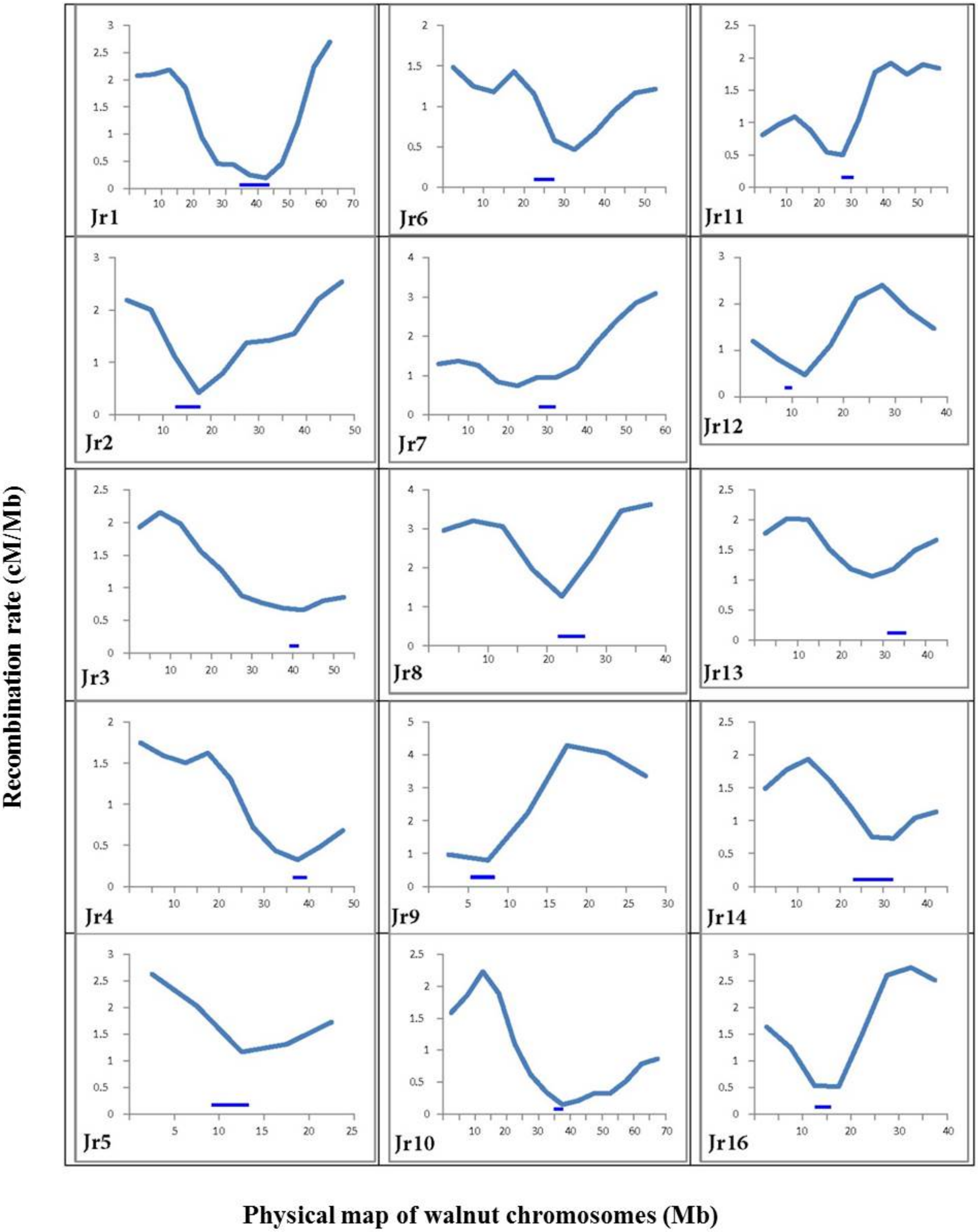

Recombination rate (cM/Mb)
Jr1
Jr6
Jr11
Jr2
Jr7
Jr12
Jr3
Jr8
Jr13
Jr4
Jr9
Jr14
Jr5
Jr10
Jr16
Physical map of walnut chromosomes (Mb)

Figure S2. Recombination rates expressed as cM/Mb across the physical maps of 15 of the 16 walnut chromosomes. The bar in each figures indicates the synteny gap across all genome comparisons. The coincidence of the synteny gap and recombination rate global minimum marks the centromerics region of the walnut chromosomes.

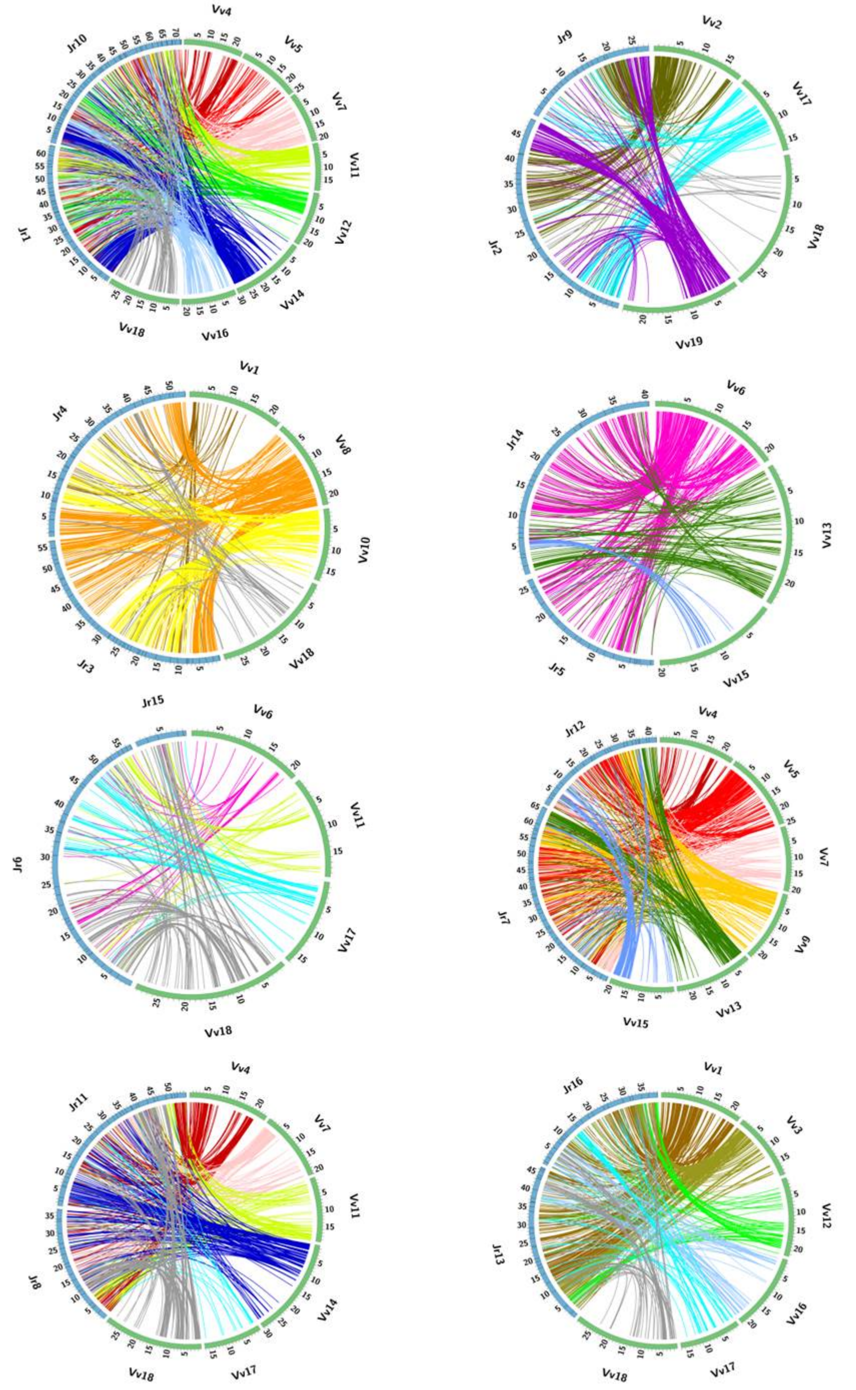

Figure S3. Relationships of the physical maps of walnut homoeologous chromosomes (blue portions of circles) to the pseudomolecules of corresponding grape chromosomes (green portions of circles). Each pseudomolecule has its unique color. A line connecting a walnut physical map

with a pseudomolecule represents a syntenic cdBES. Note that bundles of lines emanating from a grape seudomolecules bifurcate to the walnut homoeologous chromosomes, reflecting duplication of the chromosome segment in the walnut genome.
