# Supplementary material for: Synteny analysis in Rosids with a walnut physical map reveals slow genome evolution in long-lived woody perennials
Source: BMC Genomics. 2015 Sep 17;16(1):707. doi: 10.1186/s12864-015-1906-5 (PMC4574618; doi:10.1186/s12864-015-1906-5)
Supplement: Additional file 6: Table S4. — Numbers and percentages of walnut cdBES collinear with genes in the grape and poplar pseudomolecules located in SBs duplicated in the walnut genome. (DOCX 19.0 kb) [file 12864_2015_1906_MOESM6_ESM.docx]

Table S4 Numbers and percentages of walnut cdBES collinearwith genes in the grape and poplar pseudomolecules and located in SBs on an indicated chromosome (query) and in SB duplicates on another walnut chromosome

| SB | Query | No. collinear cdBES in the query | % query collinear cdBES SB not duplicated anywhere in the walnut genome | Chrom. with the highest no cdBES located in duplicated SB(s) | % collinear cdBES in duplicated  SB | Chrom. with the second highest no. cdBES located in duplicated SB(s) | % collinear cdBES in duplicated  SB |
| --- | --- | --- | --- | --- | --- | --- | --- |
| Jr-Vv | Jr1 | 342 | 41.2 | Jr10 | 49.7 | Jr6 | 7.9 |
|  | Jr2 | 267 | 24.3 | Jr9 | 68.5 | Jr11 | 3.4 |
|  | Jr3 | 276 | 35.1 | Jr4 | 60.5 | Jr8 | 2.9 |
|  | Jr4 | 151 | 13.9 | Jr3 | 83.4 | Jr6 | 2.6 |
|  | Jr5 | 81 | 2.5 | Jr14 | 88.9 | Jr6 | 8.6 |
|  | Jr6 | 160 | 48.1 | Jr15 | 18.1 | Jr1 | 11.3 |
|  | Jr7 | 360 | 15.0 | Jr12 | 78.9 | Jr14 | 2.8 |
|  | Jr8 | 200 | 19.0 | Jr11 | 72.5 | Jr1 | 7.0 |
|  | Jr9 | 176 | 2.3 | Jr2 | 85.2 | Jr12 | 6.3 |
|  | Jr10 | 240 | 28.3 | Jr1 | 60.4 | Jr8 | 5.4 |
|  | Jr11 | 308 | 18.8 | Jr8 | 72.4 | Jr1 | 6.5 |
|  | Jr12 | 286 | 7.0 | Jr7 | 84.6 | Jr12 | 5.9 |
|  | Jr13 | 320 | 14.1 | Jr16 | 75.9 | Jr7 | 6.3 |
|  | Jr14 | 253 | 53.0 | Jr5 | 29.6 | Jr7 | 7.1 |
|  | Jr15 | 45 | 13.3 | Jr6 | 62.2 | Jr8 | 24.4 |
|  | Jr16 | 277 | 27.4 | Jr13 | 54.9 | Jr9 | 6.1 |
|  | Mean | 234 | 22.7 |  | 65.4 |  | 7.2 |
| Jr-Pt | Jr1 | 308 | 26.6 | Jr10 | 67.5 | Jr6 | 5.8 |
|  | Jr2 | 266 | 31.6 | Jr9 | 65.4 | Jr3 | 2.3 |
|  | Jr3 | 261 | 29.1 | Jr4 | 67.0 | Jr2 | 2.3 |
|  | Jr4 | 172 | 16.3 | Jr3 | 82.6 | Jr2 | 1.2 |
|  | Jr5 | 115 | 5.2 | Jr14 | 77.4 | Jr16 | 7.8 |
|  | Jr6 | 176 | 64.2 | Jr16 | 8.5 | Jr11 | 6.8 |
|  | Jr7 | 336 | 8.9 | Jr12 | 74.1 | Jr11 | 14.9 |
|  | Jr8 | 176 | 29.0 | Jr11 | 52.3 | Jr6 | 10.8 |
|  | Jr9 | 184 | 11.4 | Jr2 | 87.5 | Jr3 | 1.1 |
|  | Jr10 | 207 | 30.9 | Jr1 | 68.1 | Jr8 | 1.0 |
|  | Jr11 | 305 | 31.5 | Jr8 | 60.7 | Jr7 | 7.2 |
|  | Jr12 | 244 | 50.0 | Jr7 | 50.0 | - | - |
|  | Jr13 | 283 | 38.9 | Jr16 | 46.1 | Jr14 | 4.6 |
|  | Jr14 | 288 | 38.5 | Jr5 | 58.0 | Jr13 | 2.4 |
|  | Jr15 | 31 | 0.0 | Jr6 | 61.3 | Jr5 | 38.7 |
|  | Jr16 | 200 | 32.5 | Jr13 | 53.5 | Jr5 | 12.0 |
|  | Mean | 222 | 27.8 |  | 61.3 |  | 7.9 |
