# Supplementary material for: Synteny analysis in Rosids with a walnut physical map reveals slow genome evolution in long-lived woody perennials
Source: BMC Genomics. 2015 Sep 17;16(1):707. doi: 10.1186/s12864-015-1906-5 (PMC4574618; doi:10.1186/s12864-015-1906-5)
Supplement: Additional file 7: Table S5. — Pairs of syntelogs and their annotations used for estimation of Ks. (DOCX 16.3 kb) [file 12864_2015_1906_MOESM7_ESM.docx]

Table S5. Pairs of syntelogs used for estimation of *K*s

| Syntelogs | Gene annotation |
| --- | --- |
| JH040L16-JH076P15 | PREDICTED: mitochondrial import inner membrane translocase subunit TIM50-like [Glycine max] |
| JH001G22-JM032L05 | hypothetical protein VITISV_023407 [Vitis vinifera] |
| JH006B10-JH039C11 | hypothetical protein PRUPE_ppa000964mg [Prunus persica] >gi\|462413220\|gb\|EMJ18269.1\| hypothetical protein PRUPE_ppa000964mg [Prunus persica] |
| JH011I07-JH061F22 | hypothetical protein PRUPE_ppa004862mg [Prunus persica] >gi\|462405693\|gb\|EMJ11157.1\| hypothetical protein PRUPE_ppa004862mg [Prunus persica] |
| JH020A11-JH044I06 | alpha-amylase [Malus domestica] |
| JH038K06-JH047M16 | Glutathione S-transferase family protein isoform 1 [Theobroma cacao] >gi\|508715792\|gb\|EOY07689.1\| Glutathione S-transferase family protein isoform 1 [Theobroma cacao] |
| JH042I03-JH017L22 | putative galacturonosyltransferase 6 [Morus notabilis] |
| JH054E20-JH034O21 | Outer arm dynein light chain 1 protein, putative isoform 2, partial [Theobroma cacao] >gi\|508724082\|gb\|EOY15979.1\| Outer arm dynein light chain 1 protein, putative isoform 2, partial [Theobroma cacao] |
| JH068M04-JH036D03 | RB1-inducible coiled-coil protein 1, putative isoform 2 [Theobroma cacao] >gi\|508698762\|gb\|EOX90658.1\| RB1-inducible coiled-coil protein 1, putative isoform 2 [Theobroma cacao] |
| JM013G12-JM054I03 | hypothetical protein PRUPE_ppa006484mg [Prunus persica] >gi\|462404937\|gb\|EMJ10401.1\| hypothetical protein PRUPE_ppa006484mg [Prunus persica] |
| JM017A05-JH011K02 | PREDICTED: uncharacterized protein LOC100245399 [Vitis vinifera] >gi\|302141891\|emb\|CBI19094.3\| unnamed protein product [Vitis vinifera] |
| JM057K12-JM009A24 | C2H2 and C2HC zinc fingers superfamily protein, putative [Theobroma cacao] >gi\|508717663\|gb\|EOY09560.1\| C2H2 and C2HC zinc fingers superfamily protein, putative [Theobroma cacao] |
| JM066L21-JH061D24 | PREDICTED: pyruvate kinase, cytosolic isozyme-like [Glycine max] |
| JH064H11-JH078M03 | conserved hypothetical protein [Ricinus communis] >gi\|223549524\|gb\|EEF51012.1\| conserved hypothetical protein [Ricinus communis] |
| JH075G18-JM039K04 | transferase, putative [Ricinus communis] >gi\|223541839\|gb\|EEF43385.1\| transferase, putative [Ricinus communis] |
