# Supplementary material for: Synteny analysis in Rosids with a walnut physical map reveals slow genome evolution in long-lived woody perennials
Source: BMC Genomics. 2015 Sep 17;16(1):707. doi: 10.1186/s12864-015-1906-5 (PMC4574618; doi:10.1186/s12864-015-1906-5)
Supplement: Additional file 8: Table S6. — Characteristics and locations of gaps larger than 8 cM in the 16 walnut linkage groups. (DOCX 16.2 kb) [file 12864_2015_1906_MOESM8_ESM.docx]

Table S6 Characteristics and locations of gaps larger than 8 cM in the walnut linkage groups (LG)

| LG | Gap start (cM) | Gap length (cM) | Percent of the LG | Gap start (Kb) | Gap end (Kb) | Gap length (Kb) | Location |
| --- | --- | --- | --- | --- | --- | --- | --- |
| 15 | 33.35 | 24.12 | 64.0 | 8833 | 7352 | 1481 | interst. |
| 9 | 31.56 | 14.44 | 20.8 | 16297 | 15388 | 910 | interst. |
| 9 | 56.31 | 13.73 | 19.8 | 22311 | 19198 | 3113 | interst. |
| 2 | 66.88 | 13.72 | 19.5 | 45656 | 41624 | 4032 | interst. |
| 16 | 29.56 | 9.95 | 16.8 | 27096 | 23672 | 3424 | interst. |
| 8 | 86.28 | 9.75 | 10.4 | 33741 | 32142 | 1599 | interst. |
| 5 | 28.11 | 9.13 | 20.0 | 9232 | 8007 | 1225 | interst. |
| 12 | 31.81 | 8.67 | 14.3 | 25173 | 21645 | 3528 | interst. |
| 9 | 8.43 | 8.43 | 12.2 | 255 | 0 | 255 | terminal |
| 1 | 20.96 | 8.21 | 10.3 | 10085 | 6035 | 4051 | interst. |
| Total |  | 129.15 |  |  |  | 23618 |  |
